# Optimization of a programmable *λ*/2-pitch optical phased array

**DOI:** 10.1515/nanoph-2023-0819

**Published:** 2024-03-08

**Authors:** Ankita Sharma, John N. Straguzzi, Tianyuan Xue, Alperen Govdeli, Fu Der Chen, Andrei Stalmashonak, Wesley D. Sacher, Joyce K. S. Poon

**Affiliations:** 28286 Max Planck Institute of Microstructure Physics, Halle (Saale), Germany; Department of Electrical and Computer Engineering, University of Toronto, Toronto, Canada

**Keywords:** silicon photonics, optical phased array, programmable photonics, optimization, crosstalk

## Abstract

A challenge in optical phased arrays (OPAs) is to achieve single-lobe emission using densely spaced emitters without incurring inter-waveguide optical crosstalk. Here, we propose to heuristically optimize the amplitude and phase of each grating antenna in an OPA to correct for optical non-idealities, including fabrication variations and inter-waveguide crosstalk. This method was applied to a silicon photonic integrated circuit with 1 mm-long gratings at 775 nm spacing for operation in a wavelength range of 1450–1650 nm. We achieved a wide two-dimensional beam-steering range of 110° × 28°, evaluated over a 127° × 47° field-of-view (FOV). Within this FOV, we measured an average sidelobe suppression of 8.2 dB and focused on average, 34.5 % of the emitted power into the main lobe. We achieved a peak sidelobe suppression of 14.5 dB and 50 % of the power concentrated in the main lobe. The approach is suitable for applications that require alias-free out-of-plane emission.

## 1 Introduction

Silicon photonics technology is enabling large-scale programmable optical circuits for applications in optical communications, sensing, and computing [1], [2], [3]. These programmable photonic integrated circuits (PICs) can perform arbitrary linear operations on input vectors of light in real-time using active control [4]. An integrated optical phased array (OPA) is an example of an application-specific programmable PIC, designed to generate out-of-plane emission patterns, such as steerable beams. An OPA consists of an array of coherent light emitters that form far-field patterns through their collective interference [5]. To effectively beamform with reduced sidelobes, the light emitters, commonly implemented as long weak gratings, must be spaced less than half a wavelength (*λ*/2) apart. However, this arrangement introduces a significant challenge: optical crosstalk between adjacent emitters.

There are several strategies to mitigate such crosstalk. One method is to design OPAs with non-uniform emitter spacing. This redistributes power to higher-order grating lobes, thus reducing side lobe intensities at the expense of the power in the main lobe [6], [7], [8]. Another technique is to introduce phase mismatch between adjacent waveguides. This has been applied to edge-emitting OPAs, which have demonstrated *λ*/2 emitter spacing, but only for one-dimensional (1D) beam steering [9], [10], [11]. For two-dimensional (2D) beam steering, a larger spacing (1.5*λ* to 3*λ*) is more practical [6]. Another approach is to insert subwavelength structures, such as Si ribbons or 2D photonic crystals, between waveguide grating antennas at a *λ*/2 pitch. However, the small feature sizes required are often incompatible with standard fabrication processes, and the achieved beam-steering ranges have been limited [12], [13], [14]. Recently, a *λ*/2-pitch edge-emitting OPA with a slab grating emitter achieved a 2D steering range of 140° × 13.5°, maintaining a high sidelobe suppression, but resulting in curved grating lines [15].

In this work, we introduce an alternative approach to compensate for the crosstalk in OPAs for 2D beam-steering. Using an OPA with programmable amplitude and phase control for each grating antenna element, we corrected for nonidealities, such as the inter-waveguide crosstalk and fabrication variation, using heuristic optimization to emit a single lobe. The grating antennas were 1 mm long and spaced at a pitch of 775 nm, satisfying the criterion for minimal sidelobes at *λ* = 1550 nm, while maintaining a large effective aperture length along the grating axis of the OPA. We demonstrate a wide two-dimensional steering range of 110° × 28°, assessed over a field of view (FOV) of 127° × 47°. Within this FOV we measured an average sidelobe suppression of 8.2 dB with an average of 34.5 % of the emitted power being focused into the main lobe. The circuit achieved a peak sidelobe suppression level of 14.5 dB while maintaining 50 % of the emitted power in the main lobe.

## 2 Operating principle

**Figure 1:**
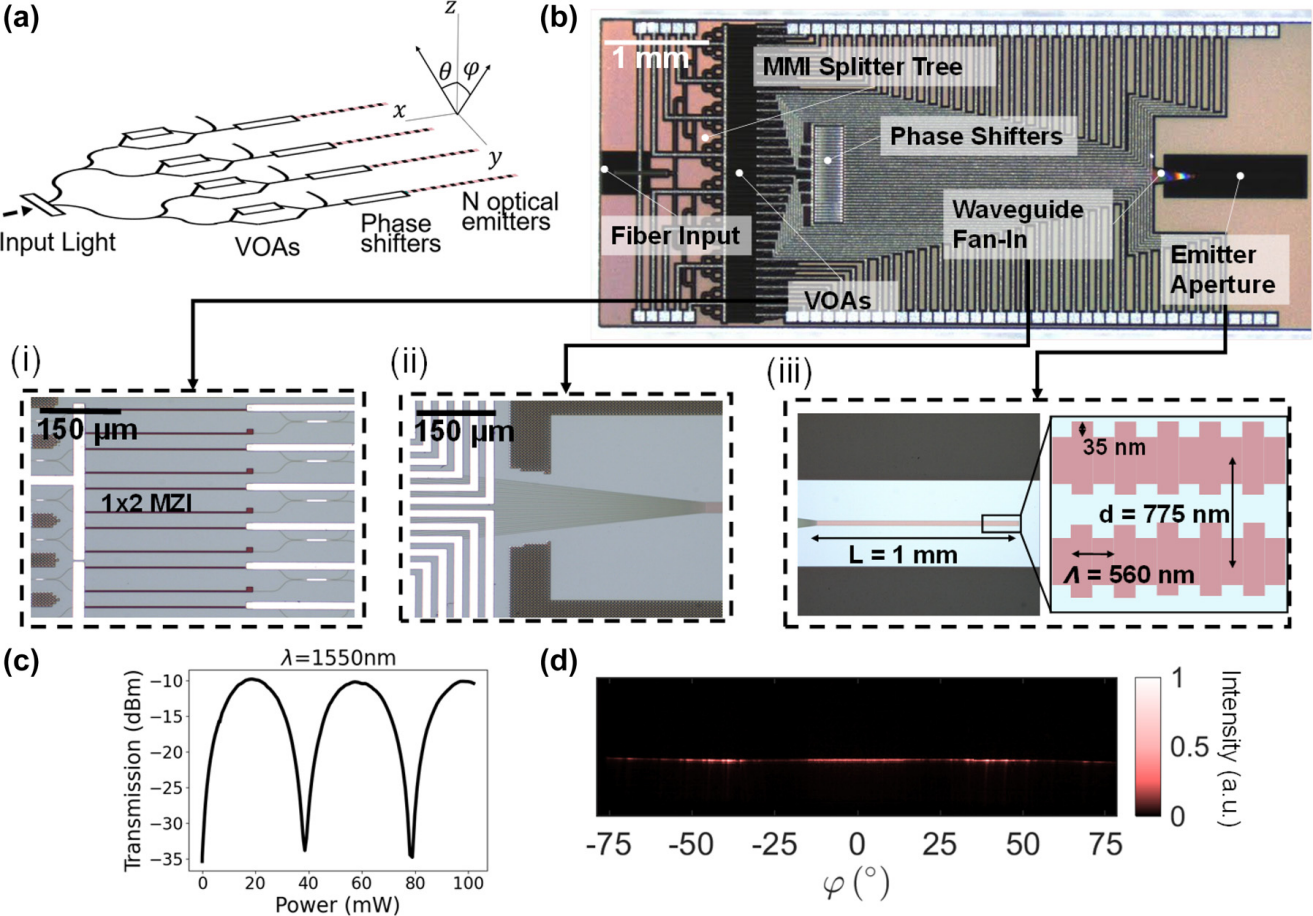
OPA circuit design. (a) Schematic of the OPA. (b) Annotated micrograph of the fabricated PIC: (i) array of variable optical attenuators (VOAs), (ii) waveguide fan-in region, and (iii) emitter aperture. (c) Measured transmission of a VOA at *λ* = 1550 nm as a function of the applied power. (d) Measured far-field of an isolated grating antenna at *λ* = 1450 nm.

In the absence of optical crosstalk, the far-field emission pattern of an OPA with identical antenna elements is modeled as the product of the far-field emission of a single antenna and the array factor, which represents the spatial arrangement and relative phase of the antennas in the array [5]. This model assumes that every antenna in the array emits an identical pattern. However, this assumption becomes inaccurate when there is significant coupling between elements in the array [16]. In this situation, to express the total far-field pattern, one can sum the average emission pattern of an individual antenna, *S* ^*a*^(*θ*, *φ*) with the individual pattern deviations, *ϵ*_*n*_(*θ*, *φ*) [16]. In a 1D uniform linear OPA with *N* emitters, this results in the modified equation,

(1) $$\begin{array}{ll} F\left(\theta ,\varphi \right) & =S^{a}\left(\theta ,\varphi \right)\underset{n}{\sum }a_{n}e^{-jnkd\sin\left(\varphi \right)}\\ & \;+\underset{n}{\sum }\epsilon_{n}\left(\theta ,\varphi \right)a_{n}e^{-jnkd\sin\left(\varphi \right)}, \end{array}$$

where *θ* and *φ* are the longitudinal and transverse angles illustrated in Figure 1(a), *k* is the wavenumber, and *d* is the uniform spacing between elements. *θ* and *φ* are defined with respect to the Cartesian *y* − *z* and *x* − *z* planes, respectively – $\sin\left(\theta \right)\approx \frac{x}{z}\;\text{and}\;\sin\left(\varphi \right)\approx \frac{y}{z}$ . *a*_*n*_ = |*a*_*n*_| exp(−*jϕ*_*n*_) is the complex amplitude and phase of each antenna. The first term of the equation gives the desired pattern, while the second term is the error or deviation from the ideal.

It follows from Eq. (1) that for any given far-field angle, [*θ*, *φ*], a set of complex coefficients, {*a*_*n*_}, can be chosen to minimize the error [16]. Implementing this concept as a PIC necessitates a programmable circuit with independent control of the amplitude and phase of each grating antenna. We can choose the normalization to constrain |*a*_*n*_| < 1. A schematic of the OPA design is shown in 1(a). In the Supplementary information, we include a brief numerical study, which demonstrates the effectiveness of this method for compensating crosstalk in 1 mm-long waveguide grating arrays, showing results for beam-forming under different array conditions.

## 3 Circuit design

### 3.1 Photonic design and fabrication

To experimentally validate the approach in Section 2, we designed a 1 × 32-element OPA with independent amplitude and phase control of each grating antenna. This OPA architecture permitted 2D beam-steering through a combination of wavelength and phase tuning. The PIC was fabricated in the Advanced Micro Foundry silicon photonics general-purpose fabrication process offered by CMC Microsystems [17]. An annotated micrograph of the fabricated circuit is shown in Figure 1(b).

At the input, light from a tunable laser was coupled into the PIC through an edge coupler and distributed to 32 channels through a series of cascaded 1 × 2 multimode interferometer (MMI) splitters, forming a binary splitter tree. The outputs of the MMI tree were modulated by an array of 32 variable optical attenuators (VOAs) followed by an array of 32 phase shifters. Both arrays were thermo-optically tuned via titanium nitride (TiN) heaters. Each TiN heater was 300 µm long and 3.5 µm wide. The power for a *π* phase shift, *P*_*π*_, was 20 mW. We used thermo-optic tuning instead of carrier-based modulators to reduce optical attenuation in the OPA.

The VOAs were balanced 1 × 2 Mach–Zehnder interferometers (MZIs), wherein thermo-optic phase shifters were integrated into both arms. Only one heater was electrically connected for tuning, while the other was included to balance the loss to achieve a high extinction ratio. Only one output of the MZI was used, and the other output was tapered to a 160 nm tip to radiate the additional power away. Measurements of the VOA test structures, as depicted in Figure 1(c), demonstrated that an extinction ratio of up to 25 dB could be attained at *λ* = 1550 nm. The phase shifter array consisted of waveguides that were laterally separated by 30 µm and thermally isolated by 15 µm-wide deep trenches, which served to minimize thermal crosstalk.

After the VOA and phase shifter arrays, the pitch of the waveguide outputs reduced from 30 µm to 775 nm (approximately *λ*/2). To limit the inter-waveguide coupling, the widths of the waveguides were varied periodically over the last 500 µm of the fan-in section. The order of waveguide widths ([500, 430, 360, 465, 395] nm) was designed so that the maximum simulated coupling between adjacent channels spaced 1 µm apart was −35 dB. The waveguide fan-in terminated in an array of 4 µm-long tapers, which transitioned into the emitter aperture.

An illustration of the waveguide grating antennas within the emitter aperture is depicted in the inset of Figure 1(b) (iii). The emitter aperture consisted of 1 mm-long waveguide grating antennas with a grating period of 560 nm and a 35 nm corrugation width to create a low-divergence beam along the *θ*-axis. The pitch of the grating antennas in the array was 775 nm, near the $\frac{\lambda }{2}$ pitch criterion. The measured far-field pattern of a single isolated grating at a wavelength of 1450 nm is shown in Figure 1(d).

### 3.2 Control electronics

**Figure 2:**
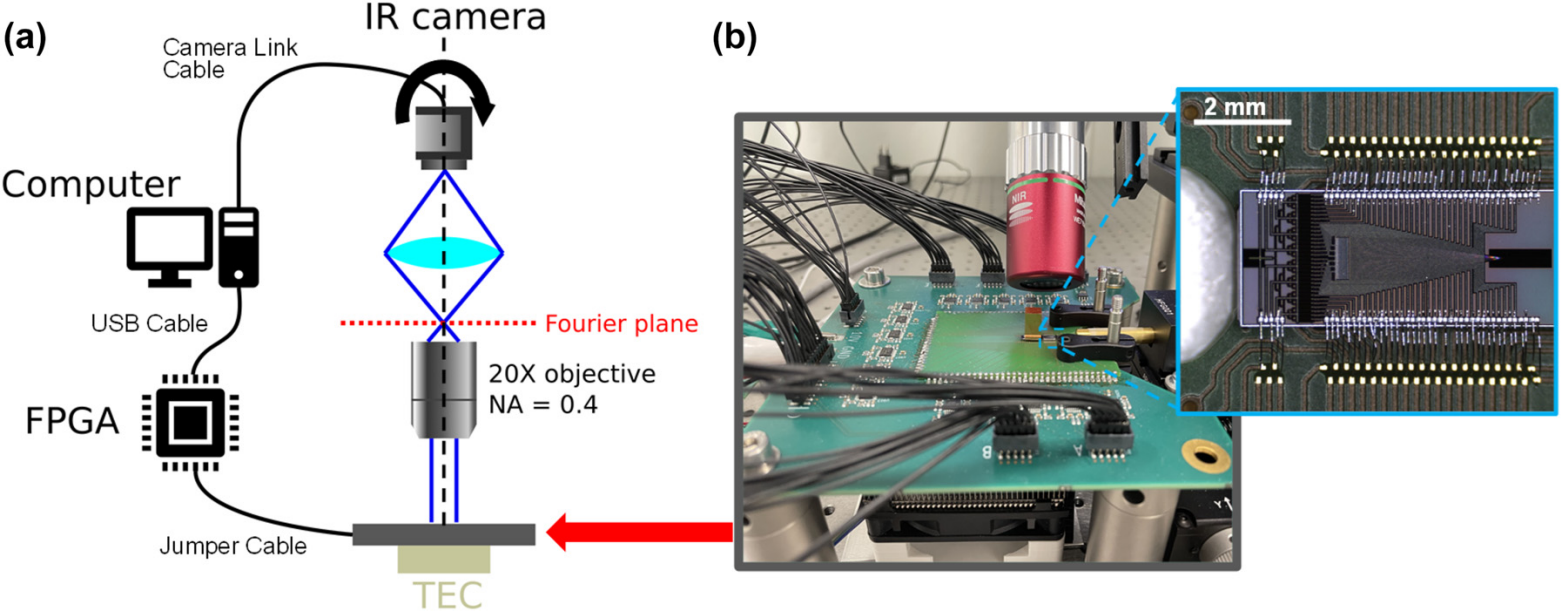
Experimental setup. (a) Schematic of the measurement setup. A rotating Fourier imaging system captures the far-field emission of the packaged OPA chip. The imaging system consists mainly of an infrared camera and a 20× objective lens (NA = 0.4). The hardware specifications are described in the main text. (b) Photograph of a packaged OPA. The PIC is wire-bonded to a printed circuit board with custom driver circuits.

The fabricated PIC was wire-bonded to a printed circuit board (PCB) and electrically controlled via a high-frequency pulse width modulation (PWM) controller from a field-programmable gate array (FPGA) (Diligent ZedBoard Zynq-7000, Model 410-248). The PWM generator was implemented using two separate frequency counters at 80 and 81 MHz, producing a repetition rate of 1 MHz with an effective number of bits of 12.5, as described in [18]. PWM values were assigned via USB to the FPGA board (2(a)). The PWM output was then filtered through an operational amplifier filter on a custom-designed driver board to generate the drive signals for the TiN heaters. Figure 2(b) shows the packaged OPA on the PCB mounted on a thermoelectric cooler (TEC) with a heat sink. The TEC-controlled holder was held at a constant temperature of 16 °C. Without temperature control, the thermal expansion of the PCB due to the heat generated by the PIC would misalign the chip relative to the fiber input. The FPGA and infrared camera were connected to a computer workstation with an Intel Xeon Gold 6428R processor at 3 GHz and 768 GB of random access memory for the optimization.

## 4 Experimental results

In practice, the values of *ϵ*_*n*_(*θ*, *φ*) in Eq. (1) are not known *apriori*. The total error is a combination of effects such as fabrication variation and thermal crosstalk in addition to optical coupling between the waveguide grating antennas [19], [20]. Therefore, we chose an optimization-driven approach to find the set of complex weights {*a*_*n*_} that minimized *ϵ*_*n*_(*θ*, *φ*) for different far-field angles. Heuristic optimization algorithms such as the genetic algorithm, particle swarm optimization algorithm, and the gradient descent algorithm are commonly used to calibrate for phase errors in OPAs caused by fabrication variation [21]. To search for solutions of {*a*_*n*_} that would form single-lobe beams, we used a combination of a multi-objective genetic algorithm and a local generalized pattern search algorithm. These algorithms were chosen because they were easy to implement and have previously been applied to array optimization [22], [23], [24].

Both algorithms required feedback on the deviation of the OPA emission pattern from the desired far-field beam pattern. To measure the far-field emission of the OPA, we used the rotatable Fourier imaging system shown in Figure 2(a). The Fourier imaging system had a 47° field-of-view (FOV) in both the *θ* and *φ* axes. Two figures of merit (FOM) were used to evaluate the far-field emission in response to a configured {*a*_*n*_}-the sidelobe suppression (SLL) and the integrated power (IP) in a region of interest surrounding the location of the desired beam [24].

**Figure 3:**
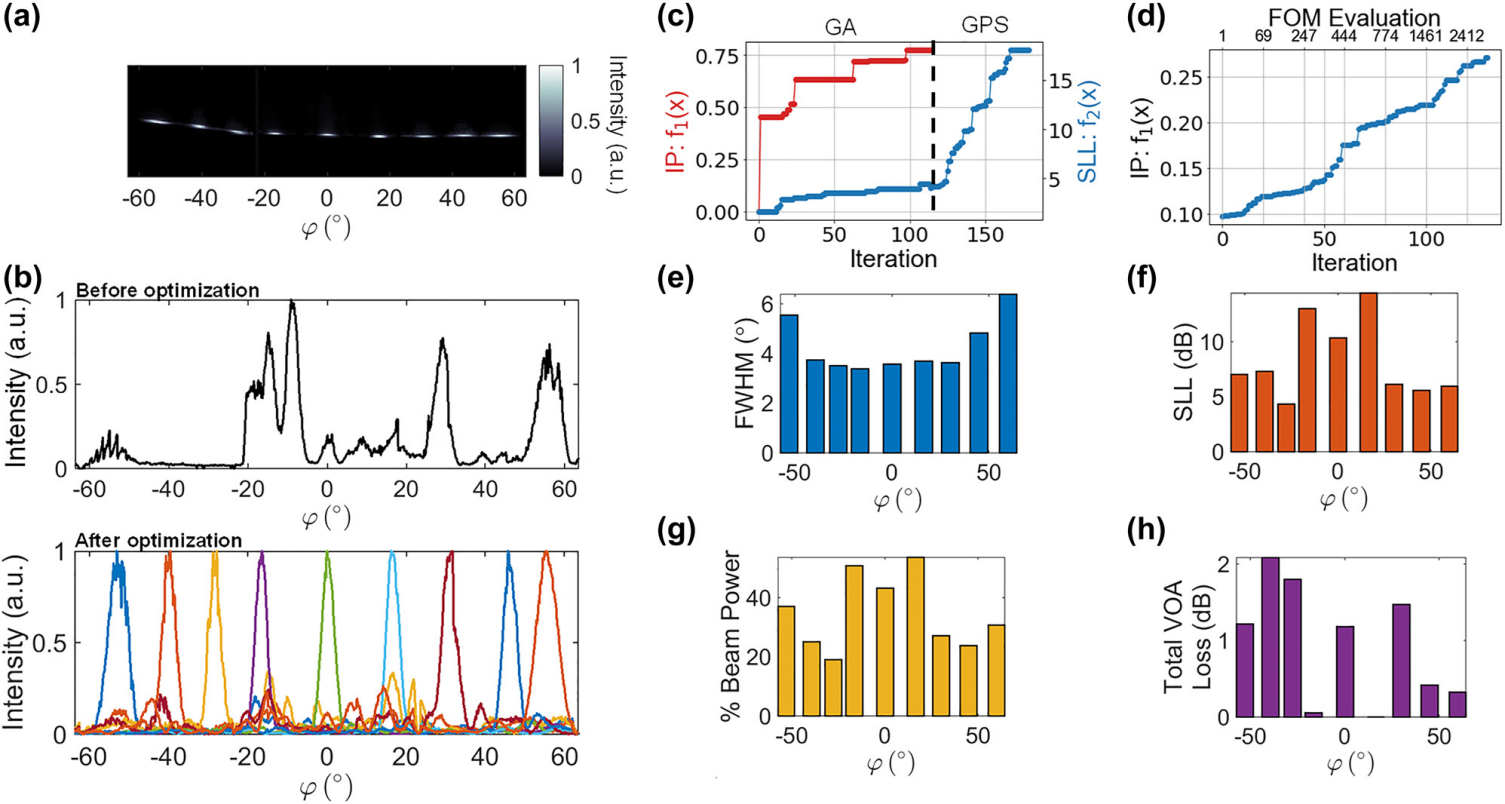
Measurement results for 9 steering angles. (a) Superimposed far-field images for different optimized angles in *φ*-axis. Each beam is normalized to its maximum intensity (b) Top: OPA emission before optimization. Bottom: Elliptical line cuts of the optimized solutions shown in (a). (c) Figures of merit (FOM) at each iteration for forming a beam at 0° without prior knowledge of existing beam solutions. A multi-objective genetic algorithm (GA) with a population size of 50 and a generalized pattern search algorithm (GPS) were used for the optimization. (d) FOM at each iteration for forming a beam at 55° with the benefit of knowing the optimized values for a solution at 50°. Only the pattern search algorithm was used for the optimization. For the various optimized angles measured: (e) FWHM, (f) sidelobe levels (g) % beam power: power in the main beam as a percentage of the total radiated power (h) total VOA loss: total radiated power normalized to the maximum observed radiated power across all measurements.

Figure 3(a) shows beams formed after optimizing for nine different angles in *φ* at a constant *λ* = 1450 nm. The OPA emission before optimization is shown in the top inset of Figure 3(b). Example training curves for the optimization procedures, which are described in more detail in the Appendix, are shown in Figure 3(c) and (d). The images in Figure 3(a) were captured with the imaging system tilted at different angles of *φ* and stitched together to measure a wider FOV. Beam-steering was achievable up to 110° in *φ*. For wide-angle characterization of the OPA, *λ* = 1450 nm was selected because it resulted in near-normal emission (*θ* ≈ −6°) so that the Fourier imaging system could rotate along *φ* while remaining fixed at *θ* = 0°. However, given that small rotations of the imaging system in *θ* were inevitable, the cross-sectional cuts in Figure 3(b) were taken along an elliptical arc to account for rotations in *θ* as described in [7].

Figure 3(e)–(h) plot the full-width at half-maximum (FWHM) beam width, sidelobe level (SLL), percentage of power in the main lobe, and total estimated VOA loss for each of the optimized beam profiles. The FWHM beam widths shown in Figure 3(e), which were approximately 3.6° between [*φ*_min_, *φ*_max_] = [−30°, 30°], were limited by the aperture size of the array. The SLL (Figure 3(f)) is a ratio of the maximum intensity in the main lobe to the maximum peak intensity outside the lobe in a 2D region defined by [*θ*_min_, *θ*_max_] = [−23.6°, 23.6°] and [*φ*_min_, *φ*_max_] = [−63.6°, 63.6°]. The power in the main lobe (Figure 3(g)) is the ratio of the integrated pixel intensity in the direction of the beam between the first nulls to the total pixel intensity in the aforementioned 2D region. Finally, the total VOA loss (Figure 3(h)) is the integrated intensity over all pixels in the 2D region normalized to the maximum total observed across all beam solutions; effectively estimating VOA loss relative to the highest power recorded within the FOV.

For all measurements, image enhancements from the infrared camera (Sensors Unlimited Inc. SU640CSX) were turned off to ensure that the pixel intensity scaled linearly with the input power. The best-optimized solution had an SLL of 14.5 dB and more than 50 % of the total power emitted in the main lobe. The other optimized solutions were limited by the emission characteristics of a single grating antenna, as depicted in Figure 1(d). For example, attempts to direct a beam at an angle of *φ* = −30° resulted in a low SLL of less than 5 dB and a beam-forming efficiency under 20 %. At this specific angle, the grating emits minimal power, which prevents the formation of a high-quality beam. Furthermore, the grating does not emit substantial power at angles >±55° , which sets the steering limits of the OPA.

**Figure 4:**
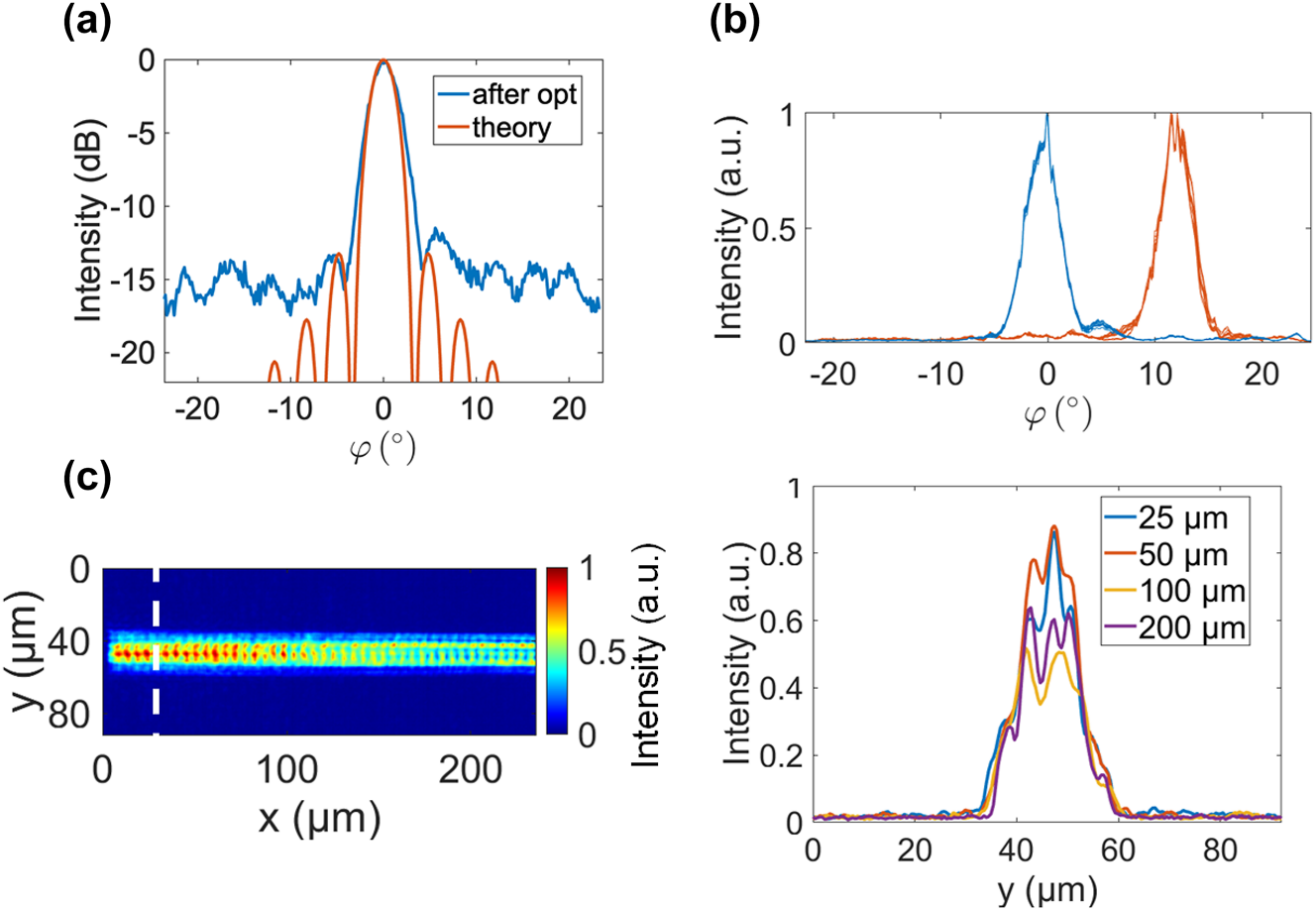
Analysis of measurement results. (a) Comparison of beam formed at 0° with diffraction pattern of a uniformly illuminated rectangular aperture. (b) Fifteen consecutive trials in which the OPA was alternately programmed using two sets of optimized coefficients, {*a*_*n*_} for *φ* = 0° and *φ* = 12.5°. (c) Left: Captured near-field emission of the OPA when the beam is steered to 0° in *φ*. Right: Vertical line cuts of the top image after propagating different distances along the grating axis. The line cuts show a persistent near-Gaussian power distribution.

Figure 4(a) shows the optimized beam profile for *φ* = 0° compared to the theoretical sinc^2^ diffraction pattern of a uniformly illuminated rectangular aperture with 32 emitters at a pitch of 775 nm. The two beam profiles show a high degree of similarity, demonstrating successful control over the crosstalk in the emitter aperture. In Figure 4(b), we investigate the robustness of two of the optimized solutions by switching between two sets of optimized coefficients, {*a*_*n*_}, for *φ* = 0° and *φ* = 12.5° fifteen times. These consecutive trials are plotted on the same figure and demonstrate that the OPA can be programmed to repeatedly form a beam at the same location with a precision of 0.15°, the resolution of the Fourier imaging system. The standard deviation of the FWHM beam widths was 0.05°. Finally, the near-field emission over the first 250 µm of the emitter aperture, depicted in Figure 4(c), revealed a persistent near-Gaussian power distribution along the grating axis. Adopting a Gaussian distribution is an effective method for sidelobe suppression in an OPA [25]. This similarity demonstrates crosstalk compensation and also suggests that the optimization process approached a near-optimal solution, in alignment with SLL being a figure of merit.

**Figure 5:**
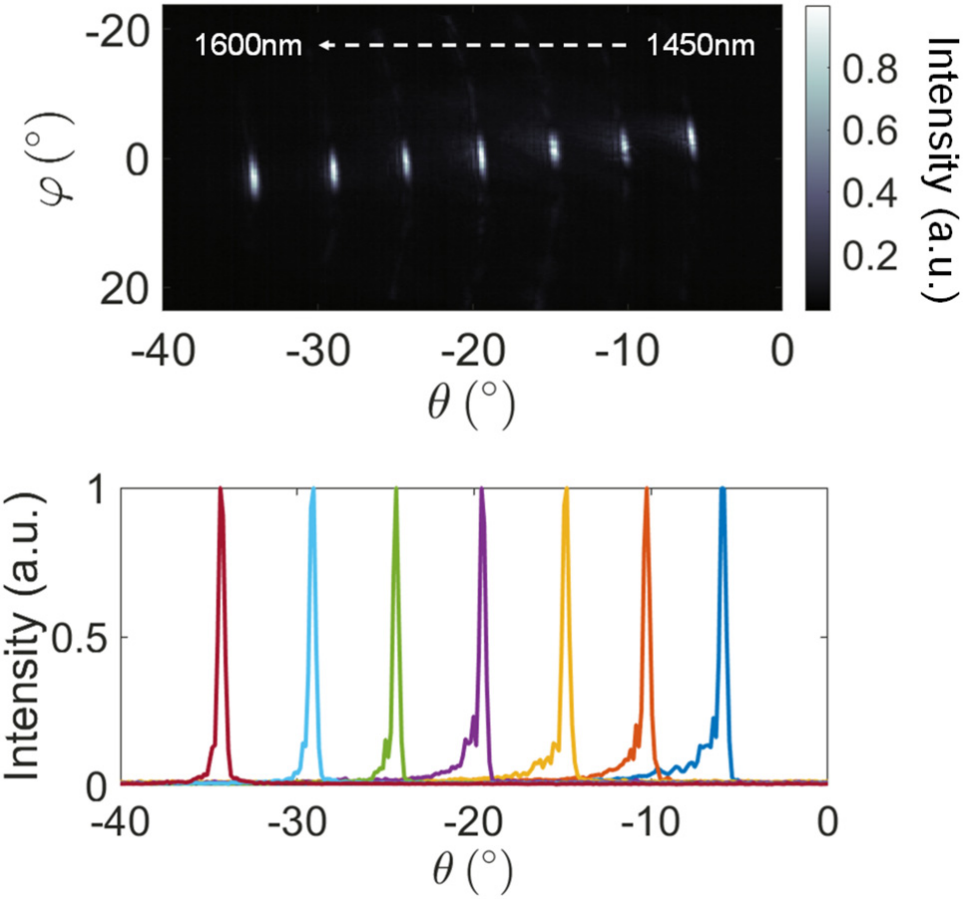
Demonstration of 2D beam-steering. Top: Superimposed far-field images for  different optimized angles in *φ*-axis and  *θ*-axis. To steer in *θ*, the wavelength is tuned from 1450 to 1600 nm. Bottom: Line cuts in *θ* of the beams in the image above.

In Figure 5, we used a combination of wavelength and phase tuning to achieve simultaneous steering in both the *θ* and *φ* directions. We traced an oblique line in the far field, by repeating the optimization process at different wavelengths and for different values of *φ*. By varying the wavelength from 1450 to 1600 nm, we demonstrated beam-steering over 28° from −6° to −34° in the *θ*-axis. The average beam divergence in *θ* (Figure 5) was 0.4°. Beam-steering in this dimension was not limited by the wavelength-dependent losses of the components in the fabricated OPA, but limited by the ability to rotate the imaging system along *θ* beyond −16°. Measurements of test structure components showed that the input wavelength of the designed OPA can be tuned from 1450 to 1650 nm.

## 5 Discussion

A limitation of our heuristic optimization approach is the long convergence time needed to find an optimal {*a*_*n*_} for beam-forming. For example, without prior knowledge of an optimized {*a*_*n*_}, in Figure 3(c) the genetic algorithm required 110 iterations to converge to a solution with approximately 50 FOM evaluations per iteration. This procedure lasted between 10 and 12 hours using the setup in Figure 2 and described in 3.2. The optimization time can be reduced to several hours when the target beam is located within <5° of a pre-existing solution at the same wavelength, and a local pattern search algorithm is used instead of a global optimization algorithm, requiring fewer FOM evaluations to converge as shown in Figure 3(d).

One evaluation of the FOM- from assigning PWM values on a PC to acquiring and analyzing a far-field image- took a total of 3 s. This time duration was constrained by the data transfer time between the FPGA and the PWM generator registers. The FPGA managed the transfer of incoming USB data to the PWM generator registers via the Advanced eXtensible Interface (AXI) bus, which allowed for updates of the 64 TiN heaters through file writes from a PC. However, data transfer through the AXI bus was performed sequentially, which created a major performance bottleneck. Using AXI Direct Memory Acess (DMA) for parallel data transfers would mitigate this bottleneck, enhancing the system’s performance and its capability to efficiently scale with an increased number of channels in the array.

The optimization algorithms also contributed to inefficiencies in the total optimization time. Specifically, the genetic algorithm often failed to find a global optimum, as illustrated in Figure 3(c), whereas the pattern search algorithm typically showed a gradual progression towards the optimal solution as seen in Figure 3(d). Both algorithms required many iterations because they were iteratively searching a high-dimensional space. For example, with 64 thermo-optic phase shifters, each with approximately 2^12.5^ possible values, there were $>10^{231}$ unique solutions of {*a*_*n*_}. Tightening the range of potential PWM values to the vicinity of [0, *V*_*π*_] for the VOAs and [0, *V*_2*π*_] for the phase shifters did not significantly reduce the search space. Transitioning to an algorithm that is more data-efficient, such as a neural network-based algorithm could improve the overall system performance and offer better scalability as the array size increases [21]. Furthermore, a deterministic or semi-deterministic approach that uses models based on the device physics would also be a more scalable solution to correct hardware errors in the circuit compared to heuristic optimization techniques [26]. With such an approach, *ϵ*_*n*_(*θ*, *φ*), could be corrected for all angles simultaneously, eliminating the requirement of a look-up table containing {*a*_*n*_} to program the OPA. We are currently investigating alternative strategies that rectify crosstalk by determining {*a*_*n*_} by matrix multiplication to achieve the desired emission pattern. Overcoming phase ambiguity within the OPA is critical.

Table 1 offers a comparison between the OPA circuit in this work and state-of-the-art OPAs that have demonstrated wide-angle beam-steering >100° . In this work, the steering range and the number of resolvable points were constrained by the capabilities of the photonic devices within the OPA. To broaden the steering range in the *φ*-axis, the waveguide grating antenna design can be optimized for a wider FWHM in *φ* [8]. Furthermore, adopting an apodized grating design would enable a more uniform emission profile across the *θ*-axis, thereby increasing the effective aperture length and consequently narrowing the beamwidth in *θ*. Increasing the aperture size along the *φ* axis can be achieved by incorporating a greater number of emitting elements. Finally, by adding amplitude in addition to phase control to each grating, we have doubled the number of heaters in the PIC compared to traditional implementations of OPAs. In the future, more efficient thermo-optic phase shifter designs could be used to reduce the power consumption of the circuit [27].

**Table 1:** Comparison of various OPA architectures for wide-angle beam steering.

Ref.	*λ* (nm)	# of emitters	Steering range (°)	% Beam power	Sidelobe suppression [dB]	Beam divergence (°)	Mechanism for wide-angle steering
[8]	1350–1630	128	140 × 19.4	3.7 %/−14.3 dB^a^	6.9	0.1 × 0.021	Aperiodic pitch
[28]	1475–1575	256	140 × 16	2.0 %/−17 dB^b^	6.0	0.051 × 0.016	Aperiodic pitch
[29]	1540–1630	64	120 × 13.9	11.0 %/−9.6 dB^b^	10.0^b^	2.72 × 0.052^b^	Aperiodic pitch
[9]	1550	64	120	72.0 %/−1.42 dB	11.4	1.6	*λ*/2-pitch detuned waveguide array
[30]	1550	16	160	Not reported	7.4	6.8	*λ*/2-pitch detuned waveguide array
[15]	1480–1580	64	140 × 13.5	High^c^	19	2.1 × 0.08^b^	*λ*/2-pitch detuned waveguide array with slab grating
This work	1450–1600	32	110 × 28	34.5 %/−4.6 dB	8.2	3.6 × 0.4	*λ*/2-pitch uniform grating array with amplitude/phase control

^a^The OPA insertion loss is not included. ^b^Evaluated for 0° solution. ^c^Exact number not given.

## 6 Conclusions

In summary, we have introduced a novel approach of using programmable amplitudes and phases to overcome the crosstalk in a 1D linear optical phased array with grating emitters spaced at approximately $\frac{\lambda }{2}$ pitch. The amplitudes and phases were found by heuristic optimization using a multi-objective genetic algorithm and a local generalized pattern search. We experimentally demonstrated crosstalk compensation in a 1 × 32-element OPA PIC with 1 mm-long waveguide grating antennas enabling a large effective aperture in the *θ*-axis. A 2D steering range of 110° × 28° was achieved respectively in the *φ* and *θ* directions, with an average beam size of 3.6° × 0.4°. Within this 2D steering range, steerable beams were repeatedly programmable. Improvements in the photonic circuit design (i.e., optimized grating antenna design, number of emitters) can increase the steering range and number of resolvable spots. Moreover, investigations into deterministic or semi-deterministic methods of compensating for the crosstalk can improve the scalability of the programmable PIC.

## Supplementary Material

Supplementary Material Details

## A Optimization procedure

Without prior knowledge of an optimized {*a*_*n*_}, to find an initial solution at a given wavelength, we used a genetic algorithm adopted from [24], which optimized the figure of merit,

(2)
$$f\left(x\right)=\max\left\{f_{1}\left(x\right),f_{2}\left(x\right)\right\},$$

where *f*_1_(*x*) is the SLL and *f*_2_(*x*) is the integrated power (IP) in a region of interest (ROI) surrounding the location of a desired beam. In our work, *x* is a vector of 64 decision variables representing the programmable amplitudes and phases in the PIC. We evaluated SLL and IP using

(3a)
$$SLL=\frac{\max\left(I\left(\theta ,\varphi \right)\right)\left\{\left(\theta ,\varphi \right)\in \text{ROI}\right\}}{\max\left(I\left(\theta ,\varphi \right)\right)\left\{\left(\theta ,\varphi \right)\notin \text{ROI}\right\}},$$

(3b)
$$IP=\frac{\iint_{\text{ROI}}I\left(\theta ,\varphi \right)\;d\sigma }{\iint I\left(\theta ,\varphi \right)\;d\sigma }.$$

Here, ROI denotes the specified region of interest surrounding the desired beam, and *I*(*θ*, *φ*) is the measured far-field intensity at the angles [*θ*, *φ*]. For the implementation of the genetic algorithm, we set the population size to 50. After applying the genetic algorithm, we optimized the solution again locally using the generalized pattern search (GPS) algorithm, which is described in [22].

After generating an initial beam, because steering should be continuous, we anticipated that {*a*_*n*_} for additional beams near the first solution could be solved using local instead of global optimization. Therefore, subsequent solutions of {*a*_*n*_} for different target beams located within <5° of the preexisting solutions in *φ* were found using only the pattern search algorithm. Unlike genetic algorithms, where new values are generated without a direct link to previous ones, pattern search maintains a consistent trajectory in the parameter space. In using GPS to find a new solution we set the objective function as *f*_2_(*x*) and then optimized a second time with *f*_1_(*x*) as the objective function if necessary.
