# Supplementary material for: Optimization of a programmable λ/2-pitch optical phased array
Source: Nanophotonics. 2024 Mar 8;13(12):2241–9. doi: 10.1515/nanoph-2023-0819 (PMC11614342; doi:10.1515/nanoph-2023-0819)
Supplement: Supplementary file 1 — Supplementary Material Details [file j_nanoph-2023-0819_suppl_001.docx]

Supplementary for:

Optimization of Programmable λ/2-pitch Optical Phased Array

*Ankita Sharma^1,2,^*, John N. Straguzzi^1^, Tianyuan Xue^1,2^, Alperen Govdeli^1,2^, Fu Der Chen*^1,2^*, Andrei Stalmashonak^1^, Wesley D. Sacher^1^ and Joyce K.S. Poon^1,2,^**

1. Max Planck Institute of Microstructure Physics, Halle 06120, Germany

2. Department of Electrical and Computer Engineering, University of Toronto, 10 King’s College Rd., Toronto, Ontario M5S 3G4, Canada

* Address correspondence to: ank.sharma@mail.utoronto.ca, [joyce.poon@mpi-halle.mpg.de](mailto:joyce.poon@mpi-halle.mpg.de)

**Numerical Study of Optimization Method**

We studied the effectiveness of our optimization method in compensating optical crosstalk through numerical simulations.

I. Transfer Matrix Model

To model the crosstalk in a 1mm-long waveguide grating array, we used a transfer matrix formalism as described in [S1]. We considered a multi-input multi-output system, which related the input to the array, represented as a complex vector of field amplitudes, $a_{in}= \left[ a_{1}, a_{2}, \ldots, a_{N} \right] ,$to its complex field outputs, $b_{out}= \left[ b_{1}, b_{2}, \ldots, b_{N} \right]$. $b_{out}$ is related to $a_{in}$ through the NxN transfer matrix C, which represents the coupling between elements in the array. In our study, we simulated the matrix C using Lumerical’s EigenMode Expansion (EME) solver. In the EME solver, we propagated a distance, L = 𝞚, in an array of N uniform waveguides, where 𝞚 is equal to the grating period of a single waveguide grating antenna. From our simulation results, we extracted an S-parameter matrix, which we then converted to a transfer matrix. Figure S1 depicts an example of a simulated coupling matrix C, as well as the associated complex field outputs calculated for both a single channel and multi-channel input excitation.


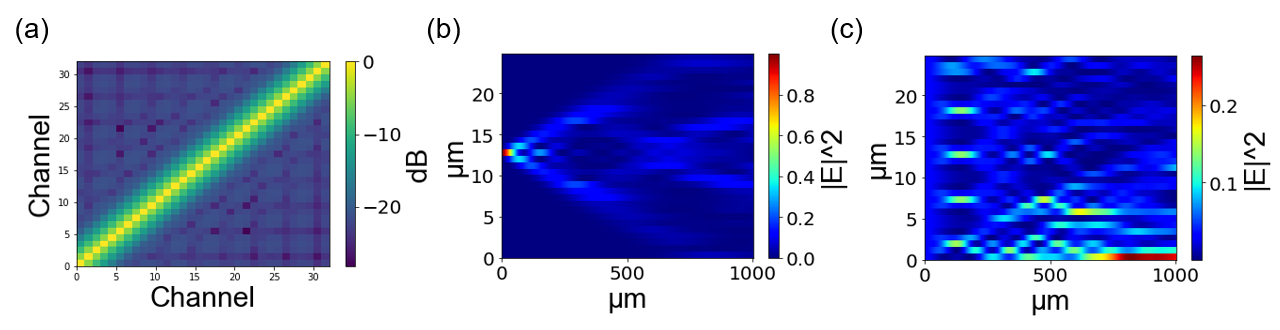


S1. (a) Simulated coupling matrix for a 32-element waveguide array after propagating 560 nm. Each waveguide cross-section is 500x220 nm, the pitch between waveguides is 775 nm and λ = 1450 nm. (b) Field amplitudes when exciting the waveguide array with a single channel. (c) Field amplitudes when exciting the waveguide array through all channels.

Next, we built in loss into our model by normalizing $b_{out}$ such that the total power decays by P(L) = P_0_ e^-2αx^, where α is equal to the grating strength of a single waveguide grating antenna. By including loss into the transfer matrix in this way we assume that the coupling constants in the matrix remain constant with loss [S1]. Figure S2 illustrates the evolution of light as it propagates in the waveguide array with α = 1.9 mm^-1^ and $a_{in}= \left[ 1, 1, \ldots, 1 \right]$. We computed the Fourier transform to obtain the far-field emission pattern of the 2D array as shown in Figure S2. This pattern is directly contrasted with the ideal far-field pattern of a uniform grating array with the same parameters in the absence of optical crosstalk.


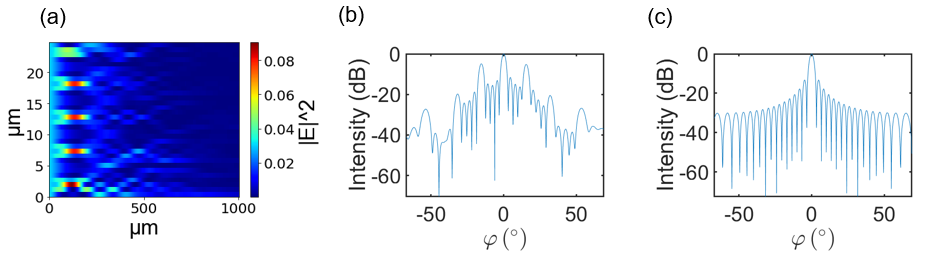


S2. (a) Field amplitudes when exciting the array in S1 through all channels and introducing loss into the transfer matrix (α = 1.9 mm^-1^). (b) Far-field emission pattern in φ-direction for array in (a). The pattern is obtained through the Fourier Transform of the 2D array. (c) Ideal far-field pattern with no crosstalk.


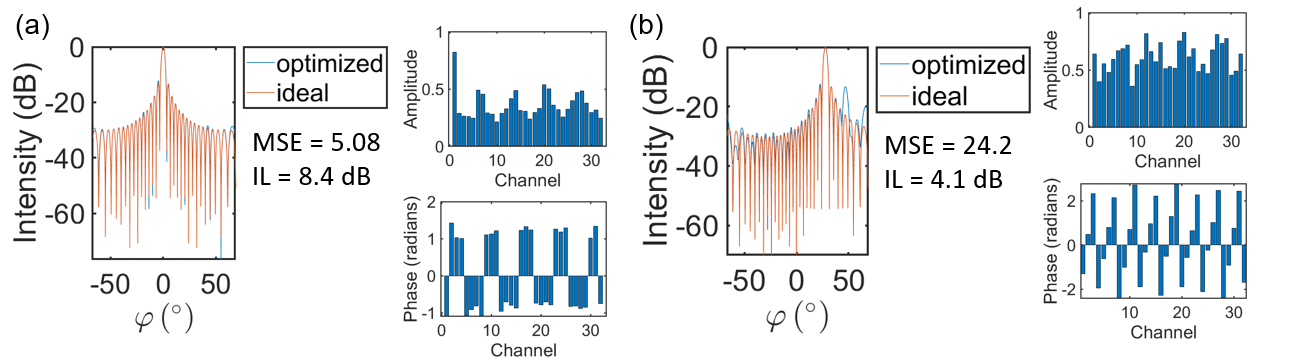


S3. Optimization results for the waveguide grating array in S2 to form (a) a beam at 0°, (b) ~30°.

II. Optimization Procedure

As described in Section 2 of the main manuscript, we can choose $a_{in}$, such that we minimize the error in the far-field pattern due to optical crosstalk. To solve for an optimal $a_{in}$ we used heuristic optimization. In Figure S3, we show the results after optimizing the input to the waveguide grating array in S2 to generate far-field beams at the angles φ = 0° and ~30°. The mean-square error (MSE) between the desired far-field pattern and the optimized pattern is calculated in log scale. In the optimization we constrain the magnitude to |$a_{N}|$<1, therefore we also report the excess incurred insertion loss (IL) for each optimized solution. For example, despite the optimized solution at 0° having a lower MSE compared to the solution at 30°, the IL is higher. Below we found optimized solutions for different levels of crosstalk, different array sizes and grating strengths at different far-field angles to test the flexibility of our approach subject to different parameters:

A. Crosstalk Levels

We tested how our method would work upon different levels of crosstalk, using coupling matrices simulated at three different wavelengths - 1450 nm, 1550 nm, and 1650 nm. We simulated a 1x32 uniform waveguide array, with waveguide cross-sections equal to 500x220 nm and a pitch between waveguides equal to 775 nm. The results of our numerical studies are shown in Figure S4. We find that our approach is robust for these different levels of crosstalk; the optimization algorithm consistently found solutions that suppressed the increased sidelobes to

≤ -13dB.


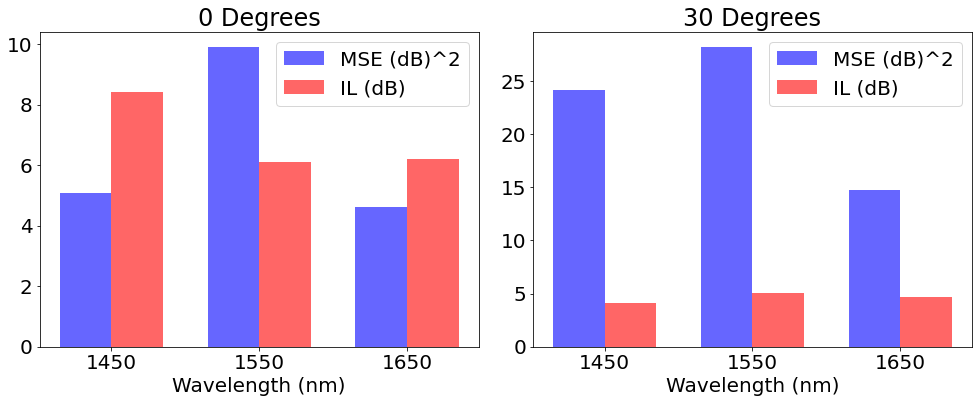


S4. Mean-square error (MSE) and insertion loss (IL) for the waveguide grating array geometry in S2 simulated at different wavelengths corresponding to different levels of crosstalk.

B. Array Size

Next, we tested if the approach would scale if the array size doubled. We find that the algorithm can still find optimal solutions that sufficiently suppresses sidelobes with similar amounts of excess incurred insertion loss to the 1x32 waveguide array (Figure S5). Nevertheless, further investigation is needed to test the limits of our method as we scale the phased array to larger sizes.


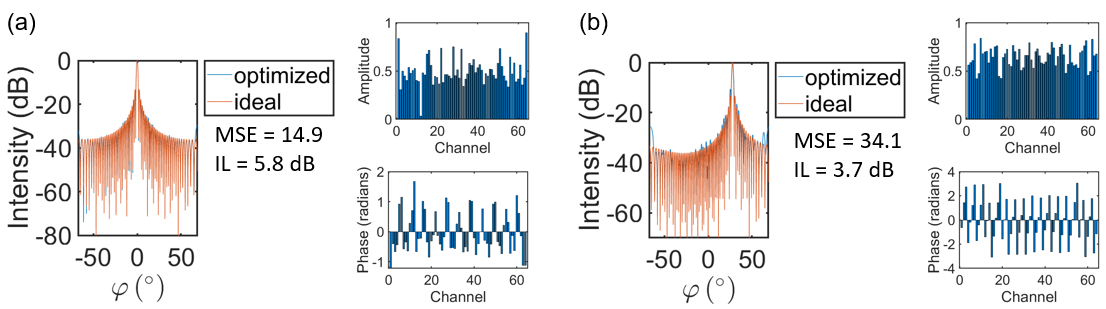


S5. Mean-square error (MSE) and insertion loss (IL) for a 1x64 uniform waveguide array to form a beam at (a) a beam at 0°, (b) ~30°. . The pitch between elements is 775 nm and λ = 1450 nm.

C. Grating Strengths

Finally, we further tested the 1x64 uniform waveguide array, for three different levels of power decay- α = 0.95 mm^-1^ 1.9 mm^-1^, 3.8 mm^-1^ . The results of these studies are shown in S5. There was little variation between the optimized solutions for these different levels of decay.


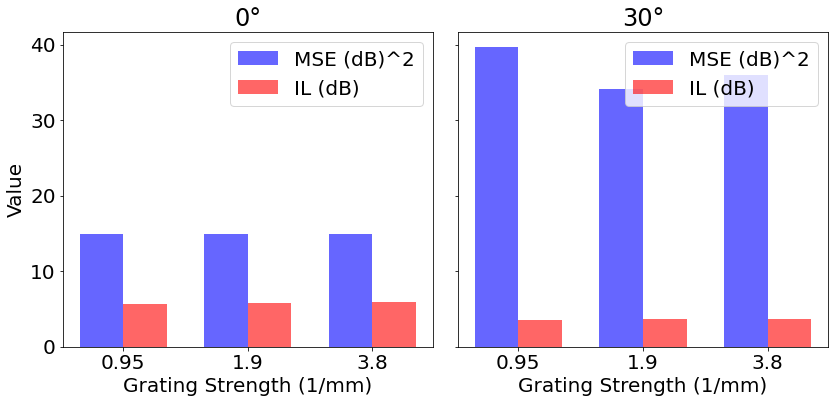


S4. Mean-square error (MSE) and insertion loss (IL) for the waveguide grating array geometry in S5 simulated for different grating strengths.

**References:**

[S1] J.K.S. Poon, P. Chak, J.M. Choi and A. Yariv, “Slowing light with Fabry-Perot resonator arrays.” *Journal of the Optical Society of America B*, Vol. 24, No.11, p.2763, Nov. 2007.
